# Fasciolosis in ruminants in Brazil

**DOI:** 10.29374/2527-2179.bjvm002924

**Published:** 2024-05-21

**Authors:** Isabella Vilhena Freire Martins, Natânia do Carmo Sperandio

**Affiliations:** 1 Veterinarian, DSc., Departamento de Medicina Veterinária, Centro de Ciências Agrárias e Engenharias (CCAE), Universidade Federal do Espírito Santo (UFES), Alegre, ES, Brazil; 2 Veterinarian, MSc., Departamento de Medicina Veterinária, (CCAE), (UFES), Alegre, ES, Brazil

**Keywords:** Brazil, control, diagnosis, epidemiology, Fasciola hepatica, Brasil, controle, diagnóstico, epidemiologia, Fasciola hepatica

## Abstract

This review aims to promote discussion about the situation of fasciolosis in ruminants in Brazil. The disease is still found more frequently in the South and Southeast regions, but reports outside these areas show the spread of the disease, including human cases. Many studies have been published on the diagnosis and control of fasciolosis, but development of field diagnosis methods and drugs that control all stages of the parasite is still a challenge. Studies should be carried out of new distribution areas and alternatives for control in Brazil, which depends on understanding the complex interactions between of the environment, ecosystems and hosts of this trematode.

## Resumo

Esta revisão tem como objetivo levantar uma discussão sobre a situação da fasciolose em ruminantes no Brasil. A enfermidade ainda se encontra mais frequentemente nas regiões Sul e Sudeste do país, entretanto relatos fora dessas regiões evidenciam a expansão da enfermidade, inclusive casos em humanos. Muitos estudos têm sido publicados sobre o diagnóstico e controle da fasciolose, entretanto, o diagnóstico a campo e fármacos que combatam todas as fases evolutivas do parasito ainda são desafios. Novos estudos devem ser realizados sobre novas áreas de distribuição e alternativas de controle em condições brasileiras, o que depende da compreensão das complexas interações entre meio ambiente, ecossistemas e hospedeiros desse trematódeo.

## Introduction

Fasciolosis in Brazil is caused by the species *Fasciola hepatica*, a parasitic trematode known commonly as liver fluke and found on all continents. It affects a variety of hosts, including domestic ruminants, wild animals and humans, and is considered a food-borne zoonosis. In ruminants, production losses are difficult to measure, but have been estimated at more than 3 billion dollars per year (Mehmood et al., 2017). In Brazil, losses were estimated at 210 million dollars between 2002 and 2011 (Molento et al., 2018).

Evidence suggests that fasciolosis is still found more frequently in the South and Southeast regions of Brazil, with some reports in other areas of the country. The prevalence of condemnation of bovine livers in slaughterhouses in these two regions has already approached 30% (Bernardo et al., 2011; Bennema et al., 2014) with some locations in the South Brazil with rates well above 50%.

Regarding human fasciolosis, there are descriptions of endemic areas in many countries. Indeed, it is classified as one of the most important neglected tropical diseases (Mas-Coma, 2020). In Brazil, 48 cases have already been officially registered, but cases are certainly underreported due to the difficulty of diagnosis (Pritsch & Molento, 2018).

The diagnosis of infections in ruminants is important and challenging, since the majority do not present clinical manifestations and the elimination of eggs occurs intermittently and in low numbers, generally not correlated with the animal's parasite load (Kahl et al., 2023). Thus, most infections are still diagnosed at the slaughterhouse, based on the observation of the parasite in the bile ducts during postmortem inspection. Studies have been carried out in several countries evaluating diagnostic tests for fasciolosis.

The limited number of drugs against *F. hepatica* available in Brazil and the difficulty in implementing management measures make it hard to control the disease. Besides this, there is growing concern about resistance to anthelmintics and their residues in meat and milk.

In this context, here we discuss the situation of fasciolosis in ruminants in Brazil, raising questions regarding epidemiology and control of the disease in the country’s varying environmental conditions.

## Biology

The definitive hosts of *F. hepatica* in Brazil include herbivorous mammals, especially cattle, buffaloes, sheep, and goats, which release eggs in feces for dispersion into the environment. Embryonation and hatching occur when eggs are exposed to light and must be in the presence of water to facilitate infection of miracidia, a ciliated larvae that must infect intermediate hosts within 24 hours after hatching (Calvani & Šlapeta, 2021).

**Figure 1:**
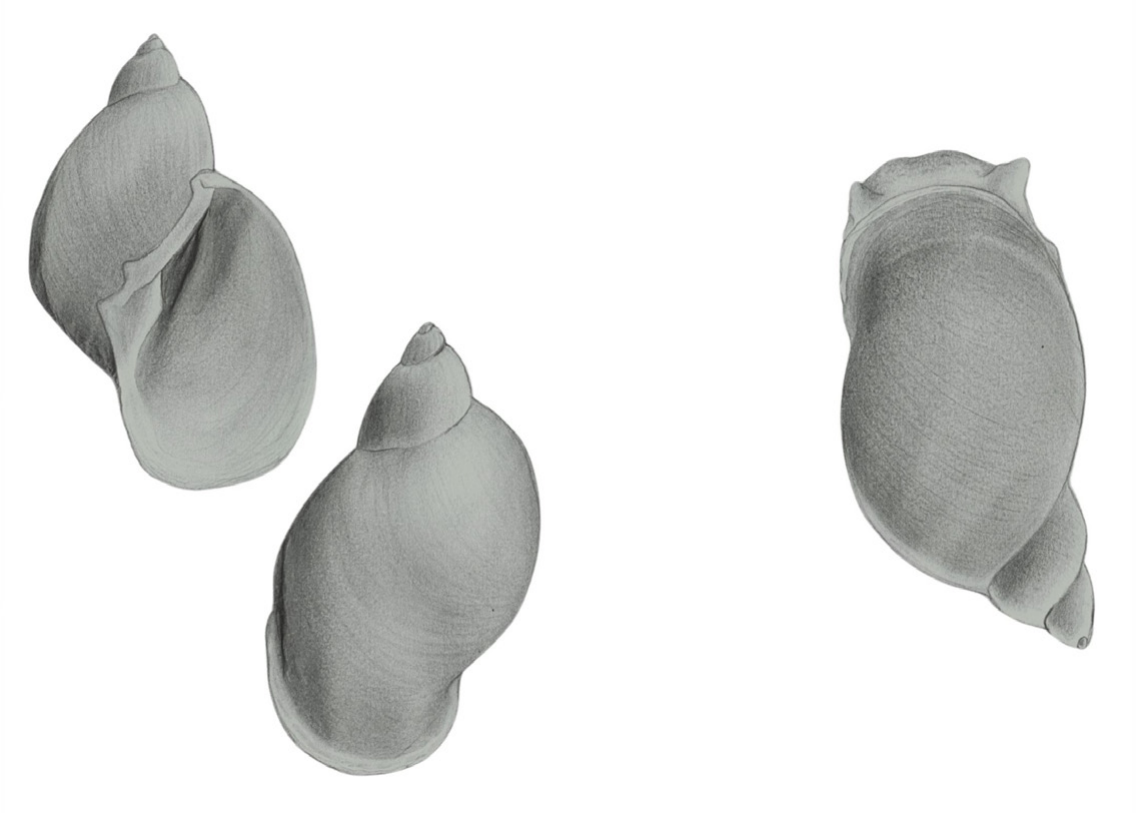
Schematic image of a specimen of *Pseudosuccinea columella*, showing convex turns to the left with turns to the right (dextrogyra).

**Source:** The authors.

The intermediate hosts are aquatic snails of the Lymnaeidae family. Other aquatic snails harboring evolutionary forms of *F. hepatica* have been reported outside Brazil (Abrous et al., 1998; Dreyfuss et al., 2002; Hamed et al., 2009). However, in Brazil, there are no reports of natural infection by other non-lymnaeid snails. Morphologically, lymnaeid are characterized by having an elongated conical shell, with convex turns, shallow or deep sutures, turns to the right (dextrogyra) and with an opening tending to be elongated-oval or rounded-ovoid, which can occupy half or even three-fourths the total shell length (Ueta, 1980) (Figure 1).

The species *Pseudosuccinea columella*, previously known in the literature as *Lymnaea columella*, is currently considered the main intermediate host of *F. hepatica* in Brazil (Bánki et al., 2024). However, reports of *F. hepatica* infection in other species of lymnaeid, such as *L. rupestris*, *Galba viatrix*, *G. cubensis* and *G. truncatula*, have been published (Medeiros et al., 2014). In recent years, studies combining molecular and morphological techniques have made it possible to identify these intermediate hosts of *F. hepatica* (Ferreira et al., 2021).

**Figure 2:**
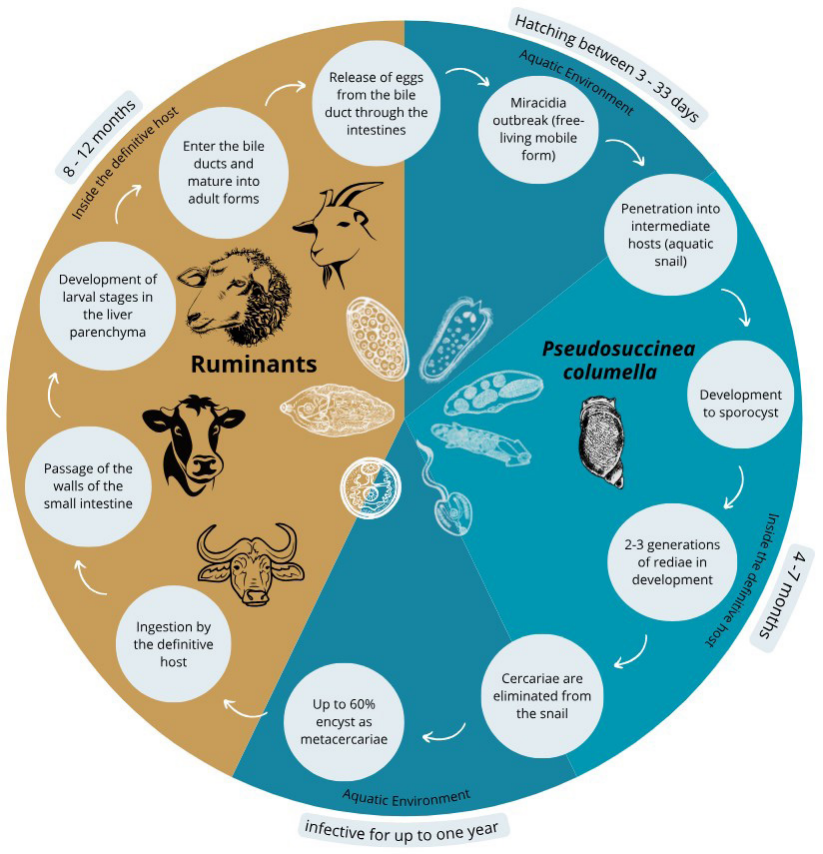
Representative scheme of the complete cycle of *Fasciola hepatica*.

**Source:** Adapted from Calvani & Šlapeta (2021).

Miracidia infect snails through active penetration through the cephalopodal mass, mantle, or tentacles, after which they migrate to the digestive gland as sporocysts. These become rediae, from which cercariae are subsequently produced. Each miracidium that enters and develops in the snail’s tissue can produce around 500 to 600 cercariae (Lunaschi, 2017), and this cercarial phase can last 45 to 60 days, involving several generations. Cercariae have tails that enable motility in water, where they encyst on nearby vegetation. After encystment, they become metacercariae, a stage that is infective to the definitive host and can remain viable in the environment for up to 12 months when humidity is maintained (Figure 2).

Once the metacercariae are ingested by the definitive host, excitation occurs in the small intestine within an hour afterward, and the juveniles penetrate the intestinal wall and head to the liver around 4 to 6 days after infection. There is an intense migration through the liver parenchyma, and after 8 to 12 weeks the adults are found in the bile ducts, where they begin laying eggs.

## Pathology and production impact

The presence of adults in the bile ducts and their migration through the epithelium results in lesions characterized by hyperplasia and calcification of bile ducts and different types of fibrosis (Tessele et al., 2013; Trivilin et al., 2014).

Macroscopic lesions in acute infection can be found, with irregular liver surface, hemorrhage and fibrosis. Peritonitis has also been described due to the migration of immature forms in the abdominal cavity and inflammation of the peritoneum. Microscopic examination can reveal extensive liver damage with hemorrhagic and necrotic spots, with the presence of immature parasites and thrombosis of the hepatic vessels (Adrien et al., 2013).

Productive losses in ruminants caused by fasciolosis include the condemnation of bovine livers, cost of treating the disease, and reduction of fertility and milk production (Howell et al., 2015; Oehm et al., 2023), in addition to death in acute cases and outbreaks (Adrien et al., 2013). Regarding weight gain, Costa et al. (2019) carried out a study in 2016 in Uruguay and confirmed loss of carcass quality in infected cattle, with the most notable weight differences being in the younger age groups. There is a lack of studies carried out in Brazil evaluating carcass or milk production losses. Dutra et al. (2010) estimated a 5.8% reduction in beef carcass weight, and Molento et al. (2018, 2020) validated models to estimate the incidence of fasciolosis and calculate the economic impact on local communities.

It is also important to highlight infections secondary to fasciolosis, since *F. hepatica* can modulate the immune system, affecting susceptibility to other pathogens, including bovine tuberculosis (Claridge et al., 2012) and clostridiosis agents (Karagulle et al., 2022).

## Distribution and epidemiology

The main hosts of *F. hepatica* in Brazil are ruminants, and cases in cattle have been reported in all states in the Midwest, South and Southeast regions of the country. Aleixo et al. (2015) also registered the presence of cattle slaughtered and condemned for fasciolosis in the states of Pará and Tocantins, and Almeida et al. (2024) reported cases in the Northeast region, confirming the expansion of this trematode throughout Brazil. The presence of *F. hepatica* in sheep, goats and buffaloes has been recorded so far in the South and Southeast regions (Carneiro et al., 2013; Pritsch et al., 2019; Serra-Freire & Nuernberg, 1992).

**Figure 3:**
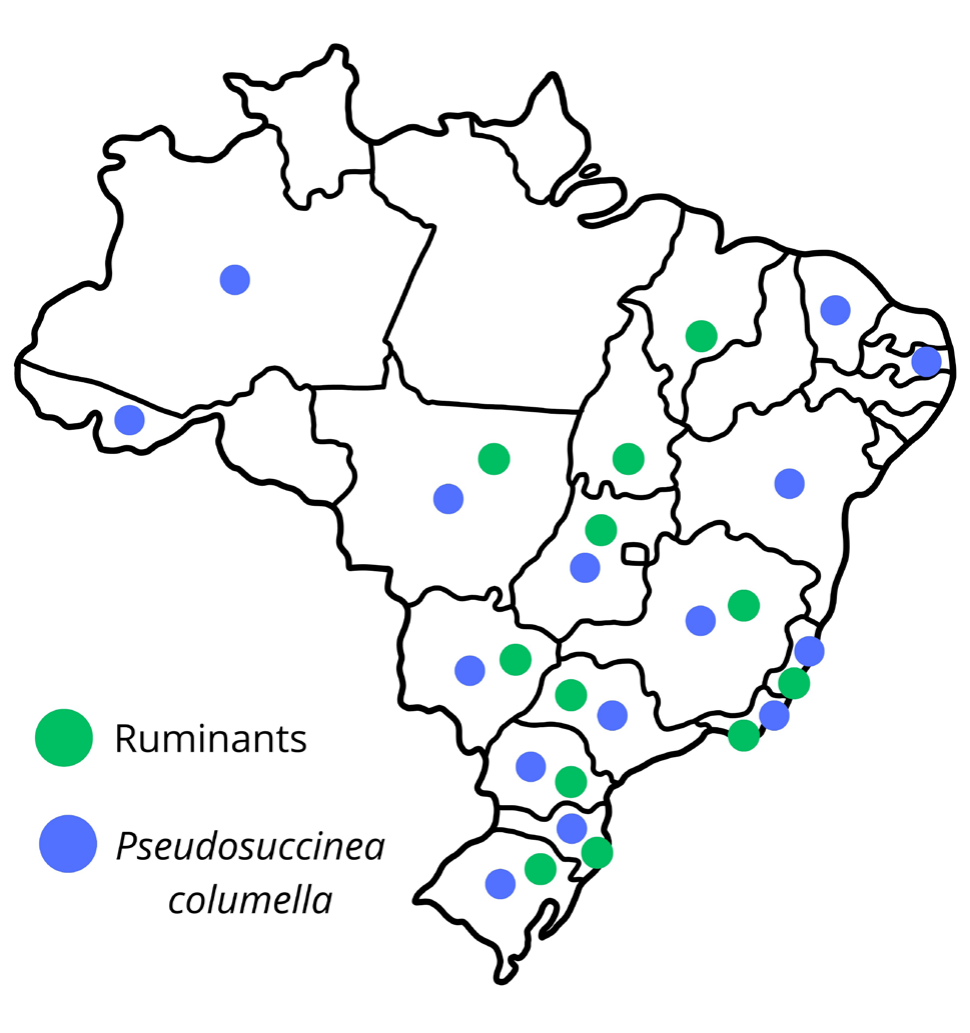
Distribution of the presence of *Fasciola hepatica* in ruminants and of *Pseudosuccinea columella* in Brazil**.**

**Source:** The authors.

Snails of the Lymnaeidae family are distributed in all Brazilian states, but the species *P. columella* is restricted to only a few states. Figure 2 indicates the Brazilian states with records of this lymnaeid and the presence of *F. hepatica* in ruminants (Figure 3).

Fasciolosis is still found more frequently in the South and Southeast regions of Brazil. According to Bennema et al. (2014), the presence of *F. hepatica* is greatest in the states of Rio Grande do Sul, Santa Catarina, São Paulo and Paraná, with records also in the states of Goiás, Mato Grosso do Sul, Mato Grosso, Minas Gerais, Espírito Santo, Rio de Janeiro and Pará.

Specific studies have been carried out by Brazilian researchers in different locations, such as Santa Catarina, with 10.81% prevalence of condemned bovine livers between 2015 and 2017 (Albuquerque et al., 2022), Rio Grande do Sul with 37.6% in 2016 and 2017 (Quevedo et al., 2018), and São Paulo with a prevalence of 7% in livers condemned due to fasciolosis between 2008 and 2011 (Mendes et al., 2019). The prevalence of condemnation of bovine livers in slaughterhouses in the south of Espírito Santo was calculated at 24.89% between 2006 and 2009 (Bernardo et al., 2011). More recently, Teixeira et al. (2023) recorded 10.4% condemnation of bovine livers in Espírito Santo and 3.6% in Rio de Janeiro. Studies of sheep and buffaloes are less common, but Cunha et al. (2007) registered a rate of 8.87% condemnation of sheep in Rio Grande do Sul. Moreover, Pritsch et al. (2019) reported an average national prevalence of 3% in buffaloes between 2003 and 2017, with averages of 11.9% in Paraná and 7.7% in Rio Grande do Sul.

Studies carried out based on sedimentation tests in animal feces have also been conducted by some authors in Brazil. For instance, in buffalos’ infection percentages of 28.37% and 23.81% were reported, respectively, in Minas Gerais (Dracz & Lima, 2014) and in Espírito Santo (Carneiro et al., 2013). In sheep and goats, the percentages of animals with *F. hepatica* eggs in their feces were 13.68 and 21.78% in Espírito Santo (Carneiro et al., 2013). In cattle, Américo et al. (2022) recorded a rate of 9.2% in Santa Catarina, Dracz & Lima (2014) identified 18.75% positive animals in Minas Gerais, and Alves et al. (2011) described prevalence of 21.33% in Espírito Santo.

The prevalence of fasciolosis is directly related to local epidemiological characteristics, especially climatic and environmental factors. Thus, its seasonality is linked to the effects of rainfall and temperature, which can directly affect the life cycle of both intermediate hosts and the parasites (Fox et al., 2011). Favorable climatic conditions in Brazil, such as high rainfall and moderate temperatures, favor the survival of snails and the spread of the larval stages of the parasite. The presence of humid and frequently flooded pasture areas encourages the development and release of cercariae present in snails (Martins et al., 2012). Bennema et al. (2014) also mentioned that in Brazil, the ideal habitat for lymnaeid snails is mainly provided by drainage or irrigation channels with slow-moving water.

Few studies in Brazil have evaluated the seasonality of snails that are intermediate hosts of *F. hepatica*. In Minas Gerais, Coelho & Lima (2003) found an increase in the number of snails in September and a reduction in December and stated that the proportion of young snails increased during the summer. In São Paulo, Amato et al. (1986) found that the population decreased between September and January and increased between March and September. In Espírito Santo, D’Almeida et al. (2016) demonstrated there are conditions for snails’ development throughout the year. The factors that influence the presence of these snails are local precipitation, seasonal water flows and water temperature. At the end of the long rainy period, the snail population tends to increase due to the oviposition of adults that were estivated during the dry season. The effect of precipitation depends on the intensity with which the rain falls, since heavy rains can lead to a reduction in the population. Temperature is also a key factor in the development and maintenance of these snails, and mild temperatures throughout the year favor their maintenance in water bodies (Oso et al., 2023).

The use of remote sensing technology and geographic information systems (GIS) has been developed to analyze the prevalence of fasciolosis by correlating climatic and environmental data. Several Brazilian studies have assessed the risk of fasciolosis using these technologies (Bennema et al., 2017; Freitas et al., 2014; Martins et al., 2012; Molento et al., 2020; Silva et al., 2020a).

Although most records of fasciolosis in Brazil are in cattle, it is worth highlighting that the conditions in endemic areas for fasciolosis in animals can be favorable for the occurrence of the disease in humans (Aleixo et al., 2015). It is necessary to evaluate the epidemiological implications in this respect to determine the impacts on a region's food security and public health.

Similarly, new molecular methods have enabled studies to monitor *F. hepatica* populations, including translocation events, allowing greater surveillance of co-infection and hybridization (Calvani & Šlapeta, 2021).

## Diagnosis

Fasciolosis can present as subclinical, acute, subacute, or chronic. Animals can suffer from anemia and jaundice, but there are no pathognomonic signs of fasciolosis. Acute fasciolosis has already been reported in Brazil in sheep (Fiss et al., 2013), goats (Carneiro et al., 2012) and cattle (Adrien et al., 2013), but generally this form of fasciolosis is responsible for death of sheep and goats, while cattle and buffaloes more often have the chronic fasciolosis.

The diagnosis is rarely made clinically, so laboratory methods are essential for *in vivo* diagnosis. The most frequent method used to diagnose *F. hepatica* eggs in feces is sedimentation. Studies using sedimentation techniques to diagnose *F. hepatica* have been carried out by various researchers in Brazil (Carneiro et al., 2018; Dias et al., 2013; Faria et al., 2008), including comparing techniques for diagnosis in feces. More recent studies have proved the good sensitivity of Flukefinder®, especially for egg counting (Kahl et al., 2023; Reigate et al., 2021).

The diagnosis of eggs in feces has drawbacks, especially low sensitivity, since elimination is intermittent, generally with a low number of eggs and still without correlation with the animal's parasite load (Kahl et al., 2023). On the other hand, several authors have argued that when choosing the technique used to detect *F. hepatica* eggs, sensitivity must be considered, along with operational factors to reduce costs and time spent, seeking a good cost-benefit ratio (Bernardo et al., 2012; Faria et al., 2008). Some authors have also argued that the simplicity and speed of the fecal sedimentation tests guarantee an advantage over others, since it can be carried out at the farm itself (Bosco et al., 2023; Reigate et al., 2021).

Serological tests are also available using blood, serum and milk, with high sensitivity and reproducibility, and high epidemiological value. However, since they detect antibodies, they do not differentiate active infections. In Brazil, Simões et al. (2017) evaluated a commercial indirect ELISA kit using animal serum and compared it to the simple fecal sedimentation test used as standard, confirming the greater sensitivity of the serological test. Coproantigens have also been validated by several authors. In Brazil, Bernardo et al. (2012) evaluated the diagnosis by coproantigens, sedimentation of feces and the presence of fasciolosis at slaughter of cattle. They found that the results obtained by the commercial ELISA kit did not differ from those obtained at slaughter, demonstrating its high sensitivity. To date, none of these serological or coproantigen tests are available in Brazil, nor have they been applied under field conditions.

The visualization of adult forms or lesions of parasitism during inspection of the liver in slaughterhouses is the most common form of diagnosis in cattle and buffaloes, which normally present the chronic fasciolosis, with fibrosis, pale livers and wall thickening of the bile duct (Tessele et al., 2013).

The differential diagnosis of fasciolosis with other diseases is also relevant. Muller et al. (2016) mentioned that due to the similarity between liver injuries in cases of chronic poisoning by *Brachiaria* spp. and cases of parasitism by *F. hepatica*, liver condemnation errors may be occurring in slaughterhouses. Still in slaughterhouses, cases of parasite migration to the lung have been reported in Brazil (Tessele et al., 2013), and should be considered for differential diagnosis or association with other diseases, as already reported by Claridge et al. (2012) and Karagulle et al. (2022).

Furthermore, it is important recognize the morphological differences between F. hepatica eggs from eggs of other trematodes that occur in Brazil, such as *Paramphistomum cervi*, since they are similar morphologically. *F. hepatica* eggs are yellow-brown and ovoid measuring 130 to 150 micrometers long by 60 to 90 micrometers wide and are different from rumen fluke egg based on color and shape following methylene blue staining. (Gordon et al., 2013).

Despite many studies addressing the diagnosis of fasciolosis, this is still a challenge considering patent infections. According to Rojas et al. (2014), there are advantages and disadvantages of methods ranging from microscopic detection of *F. hepatica* eggs in feces, to immunological diagnostic methods and more modern techniques, such as molecular tools for the specific detection of *Fasciola* DNA.

## Control

Control of fasciolosis depends on multiple factors, such as the type of farm, purpose of breeding, herd management, and products available for host treatment. Integrated control is important, combining measures that reduce intermediate hosts and control infection in definitive hosts (Carmona & Tort, 2017). Especially in ruminants, the identification of risk areas where animals become infected, such as flooded areas or areas at risk of flooding, is an essential measure to help control fasciolosis. Thus, the possibility of restricting animals in these areas is important for medium to long-term control.

The adoption of measures to control snails is essential to reduce cases in endemic areas, and currently the use of chemicals for control in these animals is discouraged due to their low selectivity and long residual period in the environment. Several researchers in Brazil have studied alternative control, using essential oils (Ito et al., 2023; Sperandio et al., 2022), entomopathogenic nematodes (Sperandio et al., 2023; Tunholi et al., 2017; Vidal et al., 2021) and even ducks and geese as a form of physical control (Ximenes et al., 1995).

Regarding the treatment of definitive hosts, there are some active ingredients available in Brazil for use in ruminants, but care must be taken to select the right product for the purpose required, to dose animals according to the product leaflet and to observe milk and meat withdrawal periods. It is common to use combination fluke and worm products, and this is an important aspect of treatment in ruminants.

According to indicators of pharmaceutical products registered in Brazil, triclabendazole, albendazole, nitroxynil, clorsulon and closantel are the active ingredients available in Brazil for the treatment of *F. hepatica* (Ministério da Agricultura Pecuária e Abastecimento, 2024). Triclabendazole is the only drug effective against early young stages (from 2 to 6 weeks), and nitroxynil and closantel are the only treatments for late young stages (from 6 weeks onwards). The other ones are against adult stages. Most fasciolicides leave undesirable residues in milk and meat, which affects food safety, so treatment should be recommended during dry periods in the case of milk cows (Imperiale & Lanusse, 2021).

Several studies have been published regarding the efficacy of flukicidal drugs in ruminants around the world, and reports are available of resistance to triclabendazole, that remains the drug of choice for treatment of larval and adult stages (Fairweather et al., 2020; Kamaludeen et al., 2019).

Efficacy tests with triclabendazole have also been conducted in Brazil, reporting success rates of 100% in goats in Espírito Santo (Carneiro et al., 2012) and in cattle in Rio Grande do Sul (Echevarria et al., 1992). This last research group conducted a study with different protocols for use in cattle and confirmed the high efficacy of triclabendazole as a fasciolicide, suggesting that it should be the drug of choice in control programs aimed at reducing the effects of fasciolosis in cattle. However, Oliveira et al. (2008), in a study in Almirante Tamandaré, Paraná, reported efficacy values of 66.3 and 57.3% respectively in sheep and goats, confirming the first case of *F. hepatica* resistance to triclabendazole in Brazil.

Studies using albendazole and albendazole sulfoxide to control *F. hepatica* were reported in the south of Espírito Santo, with activity of 97.06% for albendazole in sheep (Carneiro et al., 2013) and 26.53% and 78. 65% of efficacy, respectively, for albendazole and albendazole sulfoxide in cattle (Leão et al., 2012).

In Espírito Santo, Leão et al. (2012) found 100% efficacy of clorsulon on cattle, while in Rio Grande do Sul, Echevarria et al. (1992) reported 100% efficacy on cattle of nitroxynil and rafoxanide.

*In vitro* studies have also been conducted to evaluate the ovicidal activity of some of these drugs. Chryssafidis et al. (2024) used eggs from cattle slaughtered in Santa Catarina and tested commercial formulations of albendazole sulfoxide, closantel, nitroxynil and triclabendazole associated with fenbendazole. In this study, only closantel did not demonstrate activity in the *in vitro* hatchability test.

*In vitro* tests have been carried out by various researchers around the world, seeking to support choices of drugs to be used routinely be breeders and contribute to the diagnosis of *F. hepatica* resistance. Some authors in Brazil have also carried out *in vitro* studies using plant extracts (Marques et al., 2020), essential oils (De Mello et al., 2023; Silva et al., 2020b) and helminthophagous fungi (Dias et al., 2013), seeking alternative forms to control *F. hepatica* adults or eggs.

## Final considerations

Ruminants are the most common hosts of fasciolosis in Brazil. Some are more frequently affected by chronic fasciolosis (cattle, buffaloes) and others by acute fasciolosis (goats and sheep). The knowledge about the real economic losses in Brazil associated with fasciolosis in ruminants is still necessary to indicate its importance to national livestock breeding, especially in dairy herds where studies are scarce in our country.

Regional epidemiological studies should be encouraged to clarify the real prevalence of the disease in different ruminants, especially in expanding areas. Studies carried out in recent years have used data from slaughterhouses and/or records of fecal exams, which suggests that the real prevalence of fasciolosis may be much higher and the problem much greater than detected by those methods. No less important are studies of the presence of *F. hepatica* in lymnaeids in Brazil, which should be encouraged to support diagnosis of endemic or expanding areas.

Diagnosis at the immunological or molecular level has been widely used in epidemiological studies and is important not only for identifying foci of fasciolosis, but also for monitoring and surveillance of populations of parasites and their intermediate hosts. However, low-cost, and field-applied diagnostics are still in demand.

Epidemiological surveillance of *F. hepatica* in Brazil to obtain risk index information in all regions, associated with the use of remote sensing and geographic information system technology are fundamental for decisions on methods to control fasciolosis, especially due to impacts of climate change.

Controlling fasciolosis involves identifying areas at risk, controlling snails and treating definitive hosts. However, there are several auxiliary measures that should be indicated, always based on epidemiological evidence from the place of occurrence, such as climatic data, prevalence of the disease, and presence of snails throughout the year, among others.

To establish good fasciolosis control programs, integrated measures are the best option, involving control of snails. However, this control has drawbacks, such as the biotic potential of using molluscicides. Promising studies have been carried out with fungi, nematodes, plant extracts and essential oils, but these methods have not yet been applied in the field. Physical control, based on draining wetlands or at least restricting animals from these areas, along with the use of waterfowl that are natural predators of snails, should be encouraged.

When treating ruminants, it is important to pay attention to available drugs against fascioliasis that are easy to apply, do not leave residues in meat or milk, and are effective against larval and adult stages. Meeting these requirements is not an easy task, especially in dairy herds, where the availability of drugs is restricted and the problem of residue in milk is unavoidable. It is always important to highlight that even the highly efficient drug may not be able to prevent liver condemnation, and that the existence of *F. hepatica* strains resistant to fasciolicides is another obstacle, including in Brazil.

With advances in molecular biology and the search for sustainable control strategies, the use of vaccines has been studied to prevent fasciolosis, although the search for parasite molecules with antigens with immunoprotective capacity continues. To date, there is no vaccine that is sufficiently effective for commercial development.

Therefore, control programs for fasciolosis in ruminants in Brazil must be based on local conditions, as they are dependent on understanding the complex interactions between of the environment, ecosystems and hosts of this trematode.
