# ERRATUM: Fasciolosis in ruminants in Brazil

**DOI:** 10.29374/2527-2179.bjvm004724

**Published:** 2024-07-17

Due to the error in Figure 2 the article “Fasciolosis in ruminants in Brazil” (DOI https://doi.org/10.29374/2527-2179.bjvm002924), published in Brazilian Journal of Veterinary Medicine, v. 46, e002924, 2024, was published with an error in Figure 2, where 4-7 months and 8-12 months were published, it would be 4-7 weeks and 8-12 weeks, respectively. As shown in the image with corrections.

On page 4, where the Figure 2 is shown as:

**Figure:**
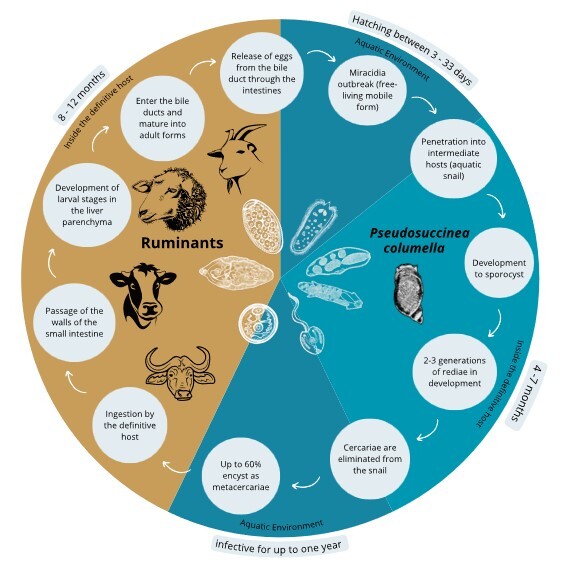


It should show:

**Figure:**
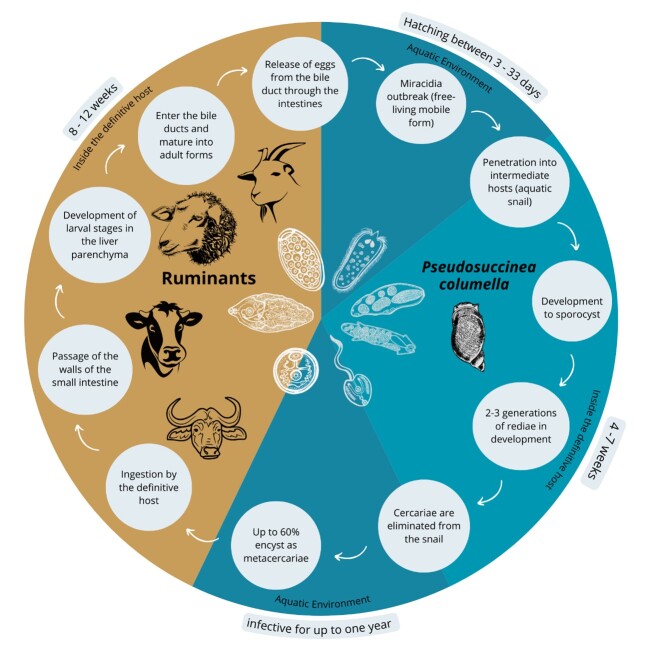


The authors apologize for the errors.

